# Graves’ Disease Following SARS-CoV-2 Vaccination: A Systematic Review

**DOI:** 10.3390/vaccines10091445

**Published:** 2022-09-01

**Authors:** Armando Patrizio, Silvia Martina Ferrari, Giusy Elia, Francesca Ragusa, Sabrina Rosaria Paparo, Valeria Mazzi, Alessandro Antonelli, Poupak Fallahi

**Affiliations:** 1 Department of Emergency Medicine, Azienda Ospedaliero-Universitaria Pisana, 56124 Pisa, Italy; 2 Department of Clinical and Experimental Medicine, University of Pisa, 56126 Pisa, Italy; 3 Department of Surgical, Medical and Molecular Pathology and Critical Area, University of Pisa, 56126 Pisa, Italy; 4 Department of Translational Research and New Technologies in Medicine and Surgery, University of Pisa, 56126 Pisa, Italy

**Keywords:** Graves’ disease, SARS-CoV-2, COVID-19, vaccine, hyperthyroidism, ASIA syndrome, autoimmune thyroid diseases

## Abstract

(1) Background: Autoimmune diseases, including autoimmune endocrine diseases (AIED), are thought to develop following environmental exposure in patients with genetic predisposition. The vaccines against severe acute respiratory syndrome coronavirus 2 (SARS-CoV-2) could represent a new environmental trigger for AIED, including Graves’ disease (GD). (2) Methods: We performed a literature search of MEDLINE/PubMed databases regarding thyroid dysfunction after SARS-CoV-2 vaccination since 1 January 2020 to 31 July 2022, considering only cases of thyrotoxicosis that meet the 2016 American Thyroid Association guidelines criteria for the diagnosis of GD and arising after administration of the anti-SARS-CoV-2 vaccine, regardless of the number of doses. (3) Results: A total of 27 articles were identified, consisting of case reports or case series, of which 24 describe the appearance of 48 new diagnoses of GD and 12 GD recurrences arising after the administration of the anti-SARS-CoV-2 vaccine, and 3 papers that instead report only 3 cases of GD relapse following vaccination. (4) Conclusions: physicians should be aware of the possibility of developing GD and other autoimmune sequelae following SARS-CoV-2 vaccination. Regardless of the underlying pathogenetic mechanisms (autoimmune/inflammatory syndrome induced by adjuvants (ASIA syndrome), cytokines induction, molecular mimicry, and cross-reactivity), an individual predisposition seems to be decisive for their development.

## 1. Introduction

As of the end of July 2022, the COVID-19 pandemic caused by SARS-CoV-2 has spread around the world with nearly 600 million cases and more than 6 million confirmed deaths, according to the WHO databases [1]. SARS-CoV-2 infection can run asymptomatically or provoke mild upper respiratory tract symptoms such as dry cough, headache, fever, and loss of smell and taste, or it can induce an interstitial pneumonia that can result in ARDS (Acute Respiratory Distress Syndrome) with the need for mechanical ventilation [2]. To date, the most effective weapon to fight SARS-CoV-2 infection is represented by primary prophylaxis with vaccines. In a short time, numerous sera have been released [3], including, for the first time, mRNA-technology-based vaccines (Pfizer-Biontech’s BNT162b2 and Moderna’s mRNA-1273) [4,5]. As with other vaccinations, the anti-SARS-CoV-2 vaccination campaign immediately highlighted the possibility of developing side effects, ranging from mild local reactions (pain at the insertion point) to systemic phenomena (fever, headache, asthenia, muscle aches) [6]. However, beyond these expected events, numerous more severe autoimmune phenomena such as myocarditis [7,8] and other manifestations have been documented soon thereafter, especially among those subjects already suffering from other forms of autoimmunity or with a familiarity for it [9]. There are several reports of auto-inflammatory and autoimmune reactions also affecting the endocrine system and the thyroid, especially in the form of subacute thyroiditis [10] and GD [11,12,13,14,15,16,17,18,19,20,21,22,23,24,25,26,27,28,29,30,31,32,33,34,35,36,37,38]. GD is an autoimmune thyroid disease characterized by the presence of autoantibodies against the TSH receptor (TRAb) expressed on thyrocytes, which cause thyroid gland growth and thyroid hormone synthesis and release with consequent hyperthyroidism [39,40]. Given the remarkable diffusion of anti-SARS-CoV-2 vaccination and its impact on public health, in this review, we report all the postvaccine cases of GD documented in the literature up to 31 July 2022, more than a year after the beginning of the immunization campaign, focusing on their main epidemiological and clinics features. Furthermore, although a causality relationship cannot be proven yet, we summarize the potential pathogenetic mechanisms that could explain the onset of autoimmunity following the anti-SARS-CoV-2 vaccination to provide up-to-date information about this emerging topic.

## 2. Materials and Methods

We performed a literature search of MEDLINE/PubMed databases regarding thyroid dysfunction after SARS-CoV-2 vaccination from 1 January 2020 to 31 July 2022. We included original articles, reviews, viewpoints, commentaries, case series and case reports, and both published and unpublished articles. The search terms, used both separately and in combination, included: “SARS-CoV-2”, “COVID19”, “thyroid”, “Graves’ disease”, “hyperthyroidism”, “autoimmune thyroid disease”, “vaccine”, “vaccination”, “thyrotoxicosis”, and “thyroiditis”.

In accordance with the 2016 guidelines of the American Thyroid Association [41], only cases of thyrotoxicosis that meet the following criteria for the diagnosis of GD and arising after administration of the anti-SARS-CoV-2 vaccine, regardless of the number of doses, were considered: (a) thyroid function tests consistent with hyperthyroidism; (b) TRAb or TSI positivity; (c) presence of thyroid scan showing high radioactive iodine uptake (RAIU); or (d) thyroid ultrasound picture of the glandular parenchyma with diffuse hypervascularization (“thyroid inferno” pattern). The findings of this review are reported in accordance with PRISMA guidelines [42].

Univariate descriptive statistics were performed. Categorical variables were analyzed using frequencies, and quantitative continuous variables were expressed the median and interquartile range (IQR).

## 3. Results

### 3.1. General Characteristics

**Figure 1:**
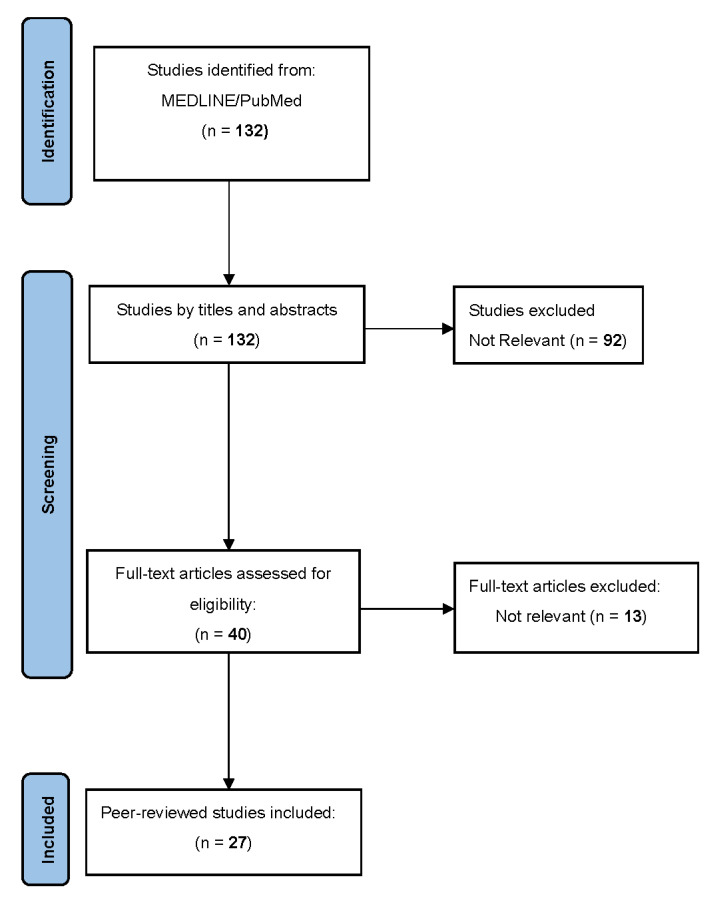
PRISMA flow diagram of study search and selection.

A total of 27 articles were identified (Figure 1) consisting of case reports or case series, of which 24 describe the appearance of 48 new diagnoses of GD and 12 GD recurrences arising after the administration of the anti-SARS-CoV-2 vaccine, and 3 papers that instead report only 3 cases of GD relapse following vaccination. All these cases have been summarized in Table 1 and Table 2 and compared in Table 3.

Among the new cases of GD, 34 are female (70.8%) and 14 men (29.2%) with a median age of 43 years [IQR 35–50.5]. Eleven women (73.3%) and four men (26.7%) with a median age of 41 [IQR 30–59] had a GD relapse. Data were collected from patients living in Europe, North America, Asia, Africa, and Oceania. Of the patients, 29/63 (46%) received the BNT162b2 vaccine (24/48 (50%) in the newly diagnosed group and 5/15 (33.3%) among relapses); 14/63 (22.2%) (13/48 (27%), and 1/15 (6.7%), respectively) received the AstraZeneca’s ChAdOx1; 5/63 (7.9%) received Moderna’s mRNA-1273; and only 2/63 (3.2%) received Janssen’s Ad26.COV2.S and CoronaVac. In 12 cases, the brand of the vaccine was not specified, but only the type (mRNA based) [38]. All cases who received more than one shot received a homologous vaccination, except for one patient who received the first two doses of Sinovac’s CoronaVac and a third booster dose of ChAdOx1 (24). One patient complained of symptoms after the third dose of mRNA-1273 [35]. Only four patients had a documented previous COVID-19 infection and in all of these patients, a new diagnosis of GD was established.

### 3.2. Clinical Features

Among those with newly diagnosed GD, only 5 of 48 patients (10.4%) reported a history of thyroid disease: 1 patient was suffering from multinodular goiter [16], 1 from hypothyroidism of unspecified etiology but on hormone replacement therapy [22], and 3 from non-GD AITD [30,34,36]. On the other hand, family history for thyroid autoimmunity was inconsistently specified. A total of 31 out of 48 patients (64.6%) in the new diagnosis group, and 8 out of 15 (53.3%) in the relapse group, developed autoimmune hyperthyroidism after a single dose of vaccine; the median time from immunization to the onset of hyperthyroidism clinical features was 10 days [IQR 5–14] and 11 days [IQR 6–28], respectively.

In patients with new-onset GD following COVID vaccination, when reported (42/48), the most frequent symptoms were palpitations (26/42, 61.9%), weight loss (15/42, 35.7%), distal tremor (12/42, 28.6%), behavioral disturbances and sleep disturbances (11/42, 26.1%), asthenia (9/42, 21.4%), gastrointestinal disturbances (6/42, 14.3%), and finally, with lower frequency, fever, exertional dyspnea, sweating, heat intolerance, and headache. The median value of measured TSH was 0.008 IU/mL (0.4–4.00) [IQR 0.004–0.01], median fT3 13.2 ng/L (2.7–5.7) [IQR 9.83–19.9], and median fT4 3.58 ng/dL (0.7–1.7) [IQR 2.5–5.34], while increased levels of TRAb were reported in 43/48 cases (89.5%) with a median value of 6.45 IU/L (0–1.5) [IQR 4.39–16.56]. A total of 4/48 (8.3%) patients were investigated only for thyroid-stimulating immunoglobulins (TSI), which turned frankly positive in all cases.

Patients affected by GD relapse, when reported (9/15), complained mainly of palpitations (77.8%), sweating (55.5%), and weight loss (33.3%); the thyroid profile showed a median TSH value of 0.01 mIU/L (0.4–4.00) [IQR 0.008–0.01], median fT3 of 13.5 ng/L (2.7–5.7) [IQR 7.97–22.45], median fT4 of 3.32 ng/dL (0.7–1.7) [IQR 1.55–3.86], and TRAb level above the normal range in all cases (100%).

In both groups, antibodies directed against thyroid antigens (thyroglobulin antibodies (TgAb) and thyroperoxidase antibodies (TPOAb)) were occasionally measured, just as an imaging examination (thyroid ultrasound or RAIU) was not always performed (Table 1 and Table 2). When available, the neck ultrasonography showed a picture of a widespread increase in gland size and vascularity, while the RAIU was high.

Given the short period of observation of these patients, all the authors reported the administration of antithyroid drugs (Thionamides) and beta-blockers as an initial therapy and symptom-control strategy for hyperthyroidism.

## 4. Discussion

Previous studies have already reported that vaccines, including those against human papillomavirus (HPV), hepatitis B (HBV), and influenza, can trigger the development or recurrence of autoimmune diseases, including chronic lymphocytic thyroiditis [43,44,45]. To respond quickly and efficaciously to the global health emergency represented by the SARS-CoV-2 pandemic, several vaccines have been approved in a short time: some of them use existing technologies, such as viral vectors (ChAdOx1, Ad26.COV2.S) [46,47] or inactivated viruses (CoronaVac) [48], but others have been based on platforms never used before, such as those based on mRNA: BNT162b2 and mRNA-1273. The latter uses a carrier system for the nucleic acid consisting of lipid nanoparticles that transfer the mRNA encoding for the antigen (SARS-CoV-2 spike protein) inside the host cells, where it is translated by the ribosomes and stimulates a robust immune response mediated by CD4 +and CD8 + T-cells [49].

The GD cases are largely collected following the administration of mRNA-based vaccines (46/63, 73%). However, it must be mentioned that these types of vaccine are the most widely administered globally. In fact, as of 11 August 2022, in the European Union, more than 1 billion shots out of approximately 1,2 billion doses administered were produced by Pfizer-Biontech and Moderna [50].

**Figure 2:**
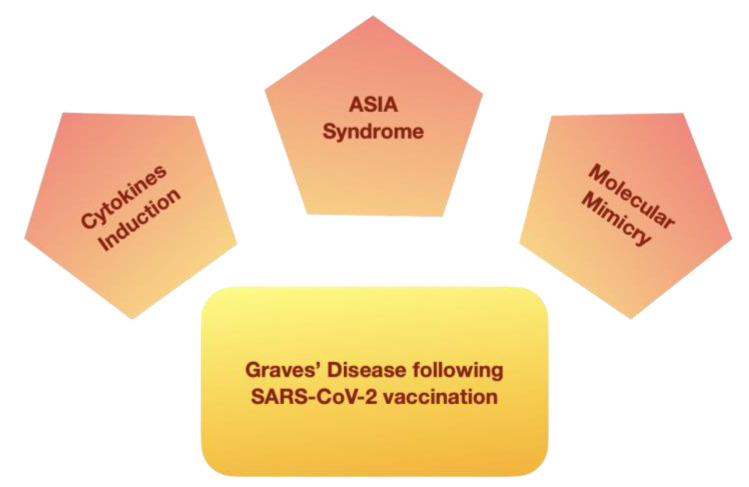
Potential pathogenetic mechanisms underlying the development of GD following SARS-CoV-2 vaccination.

Several pathogenetic mechanisms have been considered to explain the development of thyroid autoimmune reactions after SARS-CoV-2 vaccination (Figure 2).

Many authors agree that these manifestations are the result of the “autoimmune/inflammatory syndrome induced by adjuvants” (ASIA), defined by Shoenfeld in 2011 [51]. According to this theory, the adjuvants contained in the vaccine with the aim of increasing their immunogenicity can activate an immunological cascade capable, in predisposed subjects, of breaking the immunological tolerance towards self-antigens. In adenovirus-based vaccines (ChAdOx1), this role could be played by buffer/oxidation inhibitor molecules (histidine) and non-ionic surfactant (polysorbate 80), while in inactivated virus vaccines (CoronaVAc), this role is played by aluminum salts [52]. Of mRNA-based vaccines (BNT162b2 and mRNA-1273), both the nucleic acid molecule itself, capable of inducing the so-called self-adjuvant effect [53], and the lipid conjugates of polyethylene glycole (PEG) [54], which stabilize the transport nanoparticles, are believed to be responsible. Moreover, PEGs have also been considered to be the culprit of hypersensitivity and anaphylaxis reactions [55]. Regarding the ASIA syndrome hypothesis, in animal models, inflammatory responses induced by the lipid nanoparticles have been described, and they were characterized by a significant neutrophil infiltrate and by the production of numerous cytokines and chemokines, including IL-1beta/IL-6 and the macrophage inflammatory protein-α and macrophage inflammatory protein-β, which in turn could trigger a sustained inflammatory response [56].

According to Sprent and King [57], the adverse effects of anti-COVID-19 vaccines are nothing more than the epiphenomenon of an important production of interferon (IFN) type 1 and thus of a concomitant activation of the immune response. Precisely these cytokines, such as IFN-alpha, IFN-gamma, and CXCL10/IP10, peculiar of Th1-type immune response, play a crucial role in the pathogenesis of autoimmune thyroid diseases, including GD and Graves’ ophthalmopathy (GO) [58,59,60,61,62].

In addition, Poma et al. recently demonstrated that thyrocytes with direct evidence of SARS-CoV-2 genome and antigens taken from patients who died of COVID-19 carry transcriptional variations of the immunity genes, resulting in an important activation of IFN type 1 (IFN alpha) and type 2 (IFN gamma) pathways, which in turn are able to induce or reactivate thyroid autoimmunity [63]. Therefore, although the above data were obtained following natural infection, it could be speculated that the initial burst in IFN-1 release induced by vaccination could also contribute to triggering autoimmune reactions in predisposed subjects, similarly to what seems to be possible after the virus entry into the cells.

A further mechanism considered plausible for the development of autoimmune reactions from the anti-SARS-CoV-2 vaccine is represented by the “molecular mimicry” and by the cross-reactivity between some SARS-CoV-2 proteins and a variety of host antigens. In fact, it has been shown that the spike protein, the nucleoprotein, and the membrane protein of SARS-CoV-2 all cross-react with thyroid peroxidase (TPO) due to the similarity and homology of peptide sequences between this thyroid enzyme and the viral proteins [64,65]. Therefore, the SARS-CoV-2 spike protein produced within the host cells to stimulate the immune response against it could induce autoimmune reactions through the molecular mimicry mechanism. However, as observed for other vaccines (HPV, influenza and HBV) [43,44,45], the development of cross reactivity between exogenous and endogenous antigens seems limited to a minority of vaccinated subjects, demonstrating that even “molecular mimicry”, such as ASIA syndrome, requires an individual predisposition, probably of genetic nature. Nevertheless, to date, no data capable of explaining or predicting this susceptibility are available.

## 5. Conclusions

The risk of developing autoimmune sequelae after vaccination remains to be defined and there are no universally accepted criteria for their diagnosis yet. In addition, the management of these phenomena is not well defined, and the standard therapies for the “sporadic” counterparts are generally adopted. Moreover, it is becoming challenging for healthcare workers to establish the pertinence to inject the next scheduled shot in patients who have suffered from debilitating autoimmune sequelae such as in some cases of severe hyperthyroidism.

After all, we support and encourage the COVID-19 vaccination campaign as a major weapon in the fight against the pandemic, but at the same time, we want to underline the importance of being vigilant for the development of any autoimmune adverse events, including GD and hyperthyroidism, in order to have a rapid diagnosis along with the proper management of affected patients.

The small number of available papers and cases does not allow a statistical interpretation of the results, and the proposed pathogenetic mechanisms are only attempts to describe the development of this immune event without proving a causal relationship. Despite these limitations, our paper provides the most updated insight into the topic.

More than a year after the beginning of the immunization campaign, in this paper, we review the new cases and relapses of GD arising after the anti-SASR-CoV-2 vaccination, and we discuss the main features of the affected patients and the most plausible pathogenetic mechanisms (ASIA syndrome, cytokines induction, molecular mimicry, and cross-reactivity): considering the small number of cases compared to millions of vaccinated, an individual predisposition seems required; furthermore, the entire literature considered here consisted of case reports or series, proving only a temporal association.

The aim of this review is to raise awareness of healthcare workers about the possibility of developing GD and other autoimmune sequelae after SARS-CoV-2 vaccination, with the hope that future studies will allow us to identify the exact underlying pathogenetic mechanisms and subjects at highest risk.

## Figures and Tables

**Table 1:** Summary of demographic, clinical, and laboratory characteristics of new Graves’s disease cases following SARS-CoV-2 vaccination in the literature.

Gender	Age	Country	Vaccine	History of COVID	Dose	Personal/Family History of AITD	Medical History	Symptoms	Days until Symptoms	TSH (mIU/L)	fT3 (ng/L)	fT4 (ng/dL)	TgAb (IU/mL)	TPOAb (IU/mL)	TRAb (IU/L)	Thyroid US	Thyroid Scan	Reference
M	52	Italy	BNT162b2	No	2nd	None	Type 2 Diabetes, Vitiligo	Weight loss, asthenia, insomnia	20	<0.004 (N: 0.4–4)	15 (N: 2.7–5.7)	5.56 (N: 0.7–1.7)	30 (N: 0–30)	21 (N: 0–10)	6.48 (N: 0–1.49)	Enlargement and hypervascularity	N/A	Patrizio [13]
F	40	Mexico	BNT162b2	Yes	1st	None	None	Nausea, vomiting, fatigue, insomnia, and palpitations	2	<0.001 (N: 0.27–4.4)	10.5 (N: 2.04–4.4)	3.57 (N: 0.93–1.71)	210 (N: 0–44)	3450 (N: 0–5.6)	16.56 (N: 0–1.75)	Enlargement and hypervascularity	N/A	V.Lastra [11]
F	28	Mexico	BNT162b2	No	1st	None	None	Anxiety, insomnia, palpitations, distal tremors	3	<0.001 (N: 0.27–4.4)	9.2 (N: 2.04–4.4)	1.84 (N: 0.93–1.71)	33 (N: 0–44)	833 (N: 0–5.6)	5.85 (N: 0–1.75)	N/A	Diffuse toxic goiter	V.Lastra [11]
M	46	Austria	BNT162b2	No	1st	None	None	None	15	N/A	5.18 (N: 2.15–4.12)	1.63 (N: 0.7–1.7)	N/A	N/A	2.9 (N: 0–1.5)	Hypoechogenic parenchyma, large anechogenic areas with increased vascularization	Patchy, inhomogenous, normal uptake	Zettining [12]
F	71	Spain	BNT162b2	No	2nd	None	N/A	Weight loss, asthenia, afib	60	<0.005 (N: 0.38–5.33)	N/A	2.3 (N: 0.54–1.24)	<0.9 (N: 0–4)	30 (N: 0–9)	3.6 (N: 0–1.75)	Enlargement and hypervascularity	Diffuse toxic goiter	Pla Peris [14]
F	42	Spain	BNT162b2	No	1st	None	N/A	Weight loss, palpitations	14	<0.005 (N: 0.38–5.33)	N/A	2.9 (N: 0.54–1.24)	N/A	2.5 (N: 0–9)	4.39 (N: 0–1.75)	Enlargement and hypervascularity	Diffuse toxic goiter	Pla Peris [14]
F	54	Spain	mRNA-1273	No	2nd	None	N/A	Weight loss, asthenia, palpitations	14	<0.005 (N: 0.38–5.33)	N/A	4.7 (N: 0.54–1.24)	55 (N: 0–4)	30 (N: 0–9)	5.1 (N: 0–1.75)	Enlargement and hypervascularity	N/A	Pla Peris [14]
F	46	Spain	BNT162b2	No	1st	None	N/A	Weight loss, palpitations, irritability	50	<0.005 (N: 0.38–5.33)	N/A	3.2 (N: 0.54–1.24)	90 (N: 0–4)	60 (N: 0–9)	3.2 (N: 0–1.75)	Enlargement and hypervascularity	N/A	Pla Peris [14]
M	32	Italy	ChAdOx1	No	2nd	None	None	Anxiety, tachycardia, palpitations	10	0.005 (N: N/A)	7.9 (N: 2–4.4)	2.96 (N: 0.6–1.12)	N/A	N/A	7.98 (N: 0–2.9)	Enlargement and hypervascularity	N/A	Di Filippo [15]
M	35	Italy	ChAdOx1	No	1st	None	None	Nausea, headache, tachycardia, palpitations, asthenia	5	<0.004 (N: N/A)	N/A	4,96 (N: 0.6–1.12)	N/A	N/A	3.2 (N: 0–2.9)	Enlargement and hypervascularity	N/A	Di Filippo [15]
F	71	USA	BNT162b2	No	2nd	GMN/None	Stage IV breast cancer in remission	Tachycardia, palpitations, fever, dizziness, distal tremors	14	<0.02 (N: 0.3–2)	N/A	7.2 (N: 0.9–1.7)	N/A	8.9 (N: 0–9)	N/A(TSI +ve)	Multinodular Goiter	N/A	Goblirsch [16]
M	32	USA	BNT162b2	Yes	1st	None	None	Palpitations, insomnia, tremors, irritability, sweating, dyspnea	10	<0.005 (N: 0.282–4)	N/A	5.41 (N: 0.84–1.62)	53 (N: 0–40)	119 (N: 0–35)	N/A (TSI +ve)	Heterogeneous thyroid with micronodules	Diffuse uptake	Hamouche [17]
M	70	Thailand	ChAdOx1	No	2nd	None	N/A	Myalgia, palpitations, exertional dyspnea	2	<0.0036 (N: 0.35–4.94)	>20 (N: 1.88–3.18)	3.19 (N: 0.7–1.48)	N/A	N/A	3.23 (N: 0–1.75)	N/A	N/A	Sriphrapradang [21]
F	38	Spain	BNT162b2	No	1st	None	Schizophrenia	Behavioral disturbance, insomnia, sweating	12	<0.008 (N: 0.35–4.95)	7.46 (N: 0.7–1.48)	2.01 (N: 0.7–1.48)	36.57 (N: 0–5.6)	3303.71 (N: 0–5.6)	12.54 (N: 0–0.7)	Reduced echogenicity, echogenic septa, hypervascularity	Diffuse toxic goiter	Pujol [19]
F	38	USA	BNT162b2	No	1st	None	None	Fever, tachycardia, GI symptoms (thyroid storm)	5	<0.008 (N: 0.45–4.5)	N/A	8.39 (N: 0.82–1.77)	N/A	1730 (N: 0–9)	32 (N: 0–1.75)	Enlargement and hypervascularity	N/A	Weintraub [23]
F	63	USA	mRNA-1273	No	1st	None	N/A (sister with LES)	Pruritic rash upper chest and neck	7	0.011 (N: 0.55–4.78)	N/A	2.4 (N: 0.9–1.8)	N/A	1149 (N: 0–9)	22 (N: 0–1.75)	Heterogeneous thyroid and hypervascularity	Diffuse uptake	Weintraub [23]
M	30	USA	BNT162b2	No	2nd	None	None (mother post- partum GD)	Weight loss, irritability, palpitations, tremors, restless sleep	28	<0.005 (N: 0.45–4.5)	N/A	1.77 (N: 0.82–1.77)	N/A	15 (N: 0–34)	N/A (TSI +ve)	N/A	N/A	Weintraub [23]
F	40	China	BNT162b2	No	2nd	Hypothyroidism/None	None	Palpitations and tachycardia	35	<0.02 (N: 0.47–4.68)	19.8 (N: 2.77–5.29)	5.17 (N: 0.7–2.19)	7.2 (N: 0–4)	239.2 (N: 0–5)	N/A (TSI +ve)	Heterogeneous thyroid and hypervascularity	Diffuse uptake	Wai Lu [22]
F	35	Australia	ChAdOx1	No	1st	None/Hyperthyroidism	None	Palpitations, hyperphagia, heat intolerance and tremors	5	<0.02 (N: 0.5–4)	>19.5 (N: 2.2–3.9)	4.97 (N: 0.77–1.55)	33 (N: 0–4.5)	>1300 (N: 0–4.5)	24 (N: 0–0.55)	Heterogeneous thyroid and hypervascularity/A		Raven [20]
F	46	South Korea	ChAdOx1	N/A	1st	None	N/A	Chest pain, dyspnea	1	0.01 (N: 0.55–4.78)	N/A	2.63 (N: 0.89–1.76)	137.5 (N: 0–115)	77.72 (N: 0–34)	6.42 (N: 0–1.75)	Diffuse Hypervascularity	Diffuse uptake	Lee [18]
F	73	South Korea	ChAdOx1	N/A	2nd	None	N/A	Weight loss, dyspnea	14	<0.008 (N: 0.55–4.78)	N/A	5.7 (N: 0.89–1.76)	N/A	41.03 (N: 0–34)	6.30 (N: 0–1.75)	Diffuse Hypervascularity	Diffuse uptake	Lee [18]
M	20	India	ChAdOx1	N/A	1st	None	None	Weight Loss, tremors	7	0.002 (N: 0.34–5.60)	N/A	N/A	N/A	N/A	2.6 (<1.22)	N/A	N/A	Chaudhary [27]
F	46	India	ChAdOx1	N/A	1st	None/AITD	None	Weight loss	10	<0.01 (N: 0.34–5.60)	N/A	N/A	N/A	N/A	>40 (<1.22)	N/A	N/A	Chaudhary [27]
F	19	India	ChAdOx1	N/A	1st	None/AITD	None	Weight loss, palpitations, hair loss	28	<0.01 (N: 0.34–5.60)	N/A	N/A	N/A	N/A	7.32 (<1.22)	N/A	N/A	Chaudhary [27]
F	37	India	ChAdOx1	N/A	1st	None/AITD	None	Weight loss, palpitations, increased defecation	14	<0.01 (N: 0.34–5.60)	N/A	N/A	N/A	N/A	4.37 (<1.22)	N/A	N/A	Chaudhary [27]
F	31	Japan	BNT162b2	N/A	2nd	None	Type 1 Diabetes	Dyspnea, sweating, diarrhea	7	<0.005 (N: 0.61–4.23)	32.5 (N: 2.3–4)	>7.77 (N: 0.9–1.7)	82 (<28)	481 (<16)	11.9 (<2.0)	Diffuse Hypervascularity	N/A	Sakai [28]
M	22	Belgium	BNT162b2	Yes	1st	None	Ulcerative Colitis and Nephrotic Syndrome	Tremors	14	<0.01 (N: 0.27–4.20)	17.7 (N: 3.10–6.8)	3.17 (N: 0.9–1.7)	N/A	N/A	3.76 (<0.55)	Heterogeneous thyroid and hypervascularity	Diffuse uptake	Manta [29]
F	44	France	BNT162b2	N/A	1st	AITD/None	N/A	None	5	<0.01 (N: N/A)	N/A	N/A	N/A	N/A	N/A(+ve)	Diffuse Hypervascularity	N/A	Bres [30]
F	45	Singapore	BNT162b2	N/A	1st	None	None	Chest pain, palpitations	4	<0.005 (N: 0.7–4.28)	N/A	3.5 (N: 0.98–1.57)	N/A	0.3 (N: N/A)	5.75 (N: <1.76)	Heterogeneous thyroid and hypervascularity	N/A	Chua [31]
F	43	Tunisia	BNT162b2	No	1st	None	None	Palpitations, sleep disorders, heat intolerance, asthenia	3	<0.002 (N: 0.38–5.33)	N/A	5.12 (N: 0.93–1.7)	N/A	N/A	3.1 (N: <1)	N/A	Diffuse uptake	Taieb [32]
M	57	Mexico	ChAdOx1	No	1st	None	None	Tremor, palpitations, weight loss, asthenia	7	<0.005 (N: 0.3–3)	N/A	4 (N: 0.6–1.2)	N/A(+ve)	N/A(+ve)	N/A	Enlargement and hypervascularity	Diffuse uptake	Cuenca [33]
F	39	Taiwan	mRNA-1273	N/A	1st	AITD/none	None	Palpitations, tremors	14	<0.0038 (N: 0.35–4.94)	N/A	1.54 (N: 0.7–1.48)	<3.0 (N: <14.4)	64.58 (N: <5.61)	42.4 (N: <10)	N/A	N/A	Shih [34]
F	59	Taiwan	ChAdOx1	N/A	1st	None/AITD	None	Dyspnea, palpitations, dizziness	14	<0.0038 (N: 0.35–4.94)	N/A	2.28 (N: 0.7–1.48)	1494.78 (N: <14.4)	<0.3 (N: <5.61)	68.7 (N: <10)	N/A	N/A	Shih [34]
F	44	Taiwan	ChAdOx1	N/A	1st	None	None	Tremors, weight loss, heat intolerance	4	<0.0038 (N: 0.35–4.94)	N/A	2.74 (N: 0.7–1.48)	2904.39 (N: <14.4)	206.64 (N: <5.61)	80.9 (N: <10)	N/A	N/A	Shih [34]
M	42	USA	mRNA-1273	N/A	3rd	None	None	Dyspnea, sleep disturbance, weight loss, asthenia, nausea, headache	2	<0.015 (N: 0.45–4.5)	N/A	5.96 (N: 0.78–2.19)	N/A	70.25 (N: <5.6)	16.1 (N: <1.75)	Heterogeneous thyroid and hypervascularity	Diffuse uptake	Singh [35]
F	68	USA	Ad26. COV2.S	N/A	1st	None	None	Atrial fibrillation	30	<0.01 (N: 0.45–4.5)	13.8 (N: 2.5–3.9)	3.6 (N: 0.78–0.6–1.3)	N/A	5.84 (N: <5.6)	14.3 (N: <1.75)	N/A	Diffuse uptake	Singh [35]
M	50	Italy	BNT162b2	N/A	1st	None/AITD	None	Asthenia, palpitations, tremors, sleep disturbance	14	0.001 (N: 0.25–0.4)	10.47 (N: 2–4)	2 (N: 0.7–1.48)	385.49 (N: <40)	529.5 (N: <10)	5 (N: <1)	Enlargement and hypervascularity	Diffuse uptake	Ruggeri [36]
M	50	Italy	BNT162b2	N/A	1st	None/AITD	None	Asthenia, palpitations, tremors, sleep disturbance	14	0.001 (N: 0.25–0.4)	10.47 (N: 2–4)	2 (N: 0.7–1.48)	385.49 (N: <40)	529.5 (N: <10)	5 (N: <1)	Enlargement and hypervascularity	Diffuse uptake	Ruggeri [36]
F	47	Turkey	BNT162b2	No	1st	None	None	Sweating, palpitations	5	<0.01 (N: 0.27–4.2)	11 (N: 2.4.4)	3.32 (N: 0.93–1.7)	320 (N: <115)	11.2 (N: <34)	22.7 (N: <1.5)	Heterogeneous thyroid and hypervascularity	N/A	Bostan [37]
M	46	Turkey	BNT162b2	No	2nd	None	None	Sweating, palpitations, weight loss	21	<0.01 (N: 0.27–4.2)	25.3(N: 2.4.4)	>7.77 (N: 0.93–1.7)	334 (N: <115)	146 (N: <34)	9.10 (N: <1.5)	Enlargement and hypervascularity	N/A	Bostan [37]
F	51	Turkey	BNT162b2	N/A	2nd	None	Type 2 Diabetes, Hypertension	Sweating, palpitations, fever	4	<0.01 (N: 0.27–4.2)	12.6 (N: 2.4.4)	3.72 (N: 0.93–1.7)	18.2 (N: <115)	12.4 (N: <34)	5.04 (N: <1.5)	Enlargement and hypervascularity	Diffuse toxic goiter	Bostan [37]
F	53	Turkey	BNT162b2	Yes	2nd	AITD/none	None	Sweating, palpitations, weight loss	7	<0.01 (N: 0.27–4.2)	8.83 (N: 2.4.4)	4.01 (N: 0.93–1.7)	1197 (N: <115)	55 (N: <34)	17.8 (N: <1.5)	Heterogeneous thyroid and hypervascularity	Diffuse uptake	Bostan [37]
F	33	China	N/A (mRNA)	No	1st	None/AITD	N/A	N/A	7	0.01 (N:N/A)	N/A	3.4 (N: 0.62–1.24)	N/A	N/A	7.3 (N: <1)	N/A	N/A	Chee [38]
F	37	China	N/A (mRNA)	No	1st	None	N/A	N/A	7	<0.01 (N:N/A)	N/A	4.6 (N: 0.62–1.24)	N/A	N/A	3.8 (N: <1)	N/A	N/A	Chee [38]
F	37	China	N/A (mRNA)	No	2nd	None	N/A	N/A	21	<0.01 (N:N/A)	N/A	5.5 (N: 0.62–1.24)	N/A	N/A	11.2 (N: <1)	N/A	N/A	Chee [38]
F	34	China	N/A (mRNA)	No	1st	None/AITD	N/A	N/A	26	0.01 (N:N/A)	23.8 (N: 3.5–6)	5.28 (N: 0.62–1.24)	N/A	N/A	32 (N: <1)	N/A	N/A	Chee [38]
F	33	China	N/A (mRNA)	No	2nd	None	N/A	N/A	9	<0.01 (N:N/A)	N/A	2.25 (N: 0.62–1.24)	N/A	N/A	4.6 (N: <1)	N/A	N/A	Chee [38]
F	43	China	N/A (mRNA)	No	2nd	None	N/A	N/A	13	<0.01 (N:N/A)	>40 (N: 3.5–6)	5.4 (N: 0.62–1.24)	N/A	N/A	6.2 (N: <1)	N/A	N/A	Chee [38]

Abbreviations: F: female; M: male; COVID-19: coronavirus disease 2019; AITD: autoimmune thyroid disease; SLE: Systemic lupus erythematosus; GD: Graves’ disease; DM: diabetes mellitus; AFib: atrial fibrillation; GI: gastro-intestinal; MMI: methimazole; N/A: not available; +ve: positive; TSH: thyrotropin; fT3: free triiodothyronine; fT4: free tiroxine; TPOAb: anti-thyroid peroxidase antibody; TgAb: anti-thyroglobuline antibody; TRAb: TSH receptor antibody; US: ultrasound.

**Table 2:** Summary of demographic, clinical, and laboratory characteristics of Graves’s disease relapses following SARS-CoV-2 vaccination in the literature.

Gender	Age	Country	Vaccine	History of COVID	Dose	Personal/Family History of AITD	Medical History	Symptoms	Days until Symptoms	TSH (mIU/L)	fT3 (ng/L)	fT4 (ng/dL)	TgAb (IU/mL)	TPOAb (IU/mL)	TRAb (IU/L)	Thyroid US	Thyroid Scan	Reference
F	71	Austria	BNT162b2	No	2nd	GD/none	N/A	Palpitations and sweating	30	N/A	11.1 (N: 2.15–4.12)	3.56 (N: 0.7–1.7)	N/A	N/A	4.2 (N: 0–1.5)	Heterogeneous thyroid and hypervascularization	Mild increased uptake	Zettining [12]
F	64	Japan	BNT162b2	No	1st	Subclinical Hyperthyroidism/none	colorectal cancer, DM, obesity	Palpitations, dyspnea, fever, legs edema	6	<0.008 (N: N/A)	23.2 (N: N/A)	3.32 (N: N/A)	N/A	N/A	33.8 (N: N/A)	Enlargement and hypervascularity	N/A	Yamamoto [26]
F	34	Belgium	BNT162b2	No	1st	GD/none	None	Tremors, sweating, weight loss, swelling of eyelids	10	<0.01 (N: 0.4–2.75)	14.3 (N: 1.95–4.23)	2.54 (N: 0.75–1.6)	N/A	N/A	>40 (N: 0–0.55)	N/A	N/A	Pierman [25]
F	30	Thailand	CoronaVac+ChAdOx1	No	3rd (ChAdOx1)	GD on MTZ/none	None	Palpitations, weight loss, increased appetite	4	0.006 (N: 0.35–4.94)	3.21 (N: 1.88–3.18)	1.29 (N: 0.7–1.48	N/A	N/A	13.4 (N: 0–1,75)	N/A	N/A	Sriphrapradang [24]
M	34	South Korea	Ad26. COV2.S	N/A	1st	GD/none	N/A	Weight loss, palpitations	14	<0.008 (N: 0.55–4.78)	N/A	2.06 (N: 0.89–1.76)	N/A	N/A	4.24 (N: 0–1.75)	Diffuse Hypervascularity	N/A	Lee [18]
M	41	Singapore	mRNA-1273	N/A	1st	GD/none	N/A	Tremors, palpitations	5	<0.001 (N: 0.7–4.28)	N/A	3.74 (N: 0.98–1.57)	N/A	N/A	3.85 (N: <1.76)	N/A	N/A	Chua [31]
F	44	Turkey	CoronaVac	No	1st	GD/none	None	Sweating, palpitations, asthenia	7	<0.01 (N: 0.27–4.2)	9.65 (N: 2.4.4)	2.67 (N: 0.93–1.7)	119 (N: <115)	284 (N: <34)	12.18 (N: <1.5)	Heterogeneous thyroid and hypervascularity	N/A	Bostan [37]
M	49	Turkey	BNT162b2	No	2nd	GD/none	None	Sweating, palpitations, tremors	30	<0.01 (N: 0.27–4.2)	13.50 (N: 2.4.4)	3.86 (N: 0.93–1.7)	236 (N: <115)	435 (N: <34)	3.01 (N: <1.5)	Diffuse Hypervascularity	N/A	Bostan [37]
F	31	Turkey	BNT162b2	No	1st	GD/none	Breast cancer	Sweating, asthenia	21	<0.01 (N: 0.27–4.2)	21.7 (N: 2.4.4)	>7.77 (N: 0.93–1.7)	11 (N: <115)	325 (N: <34)	19.3 (N: <1.5)	Diffuse Hypervascularity	N/A	Bostan [37]
M	59	China	N/A (mRNA)	No	1st	GD/AITD	N/A	N/A	21	<0.01 (N:N/A)	N/A	3.8 (N:0.62–1.24)	N/A	N/A	12.8 (N: <1)	N/A	N/A	Chee [38]
F	74	China	N/A (mRNA)	No	2nd	GD/AITD	N/A	N/A	11	0.02 (N:N/A)	N/A	1.08 (N:0.62–1.24)	N/A	N/A	6.2 (N: <1)	N/A	N/A	Chee [38]
F	25	China	N/A (mRNA)	No	2nd	GD/AITD	N/A	N/A	11	0.01 (N:N/A)	6.3 (N: 3.5–6)	1.16 (N:0.62–1.24)	N/A	N/A	2.9 (N: <1)	N/A	N/A	Chee [38]
F	41	China	N/A (mRNA)	No	2nd	GD/none	N/A	N/A	28	<0.01 (N:N/A)	N/A	3.88 (N:0.62–1.24)	N/A	N/A	3.9 (N: <1)	N/A	N/A	Chee [38]
F	24	China	N/A (mRNA)	No	2nd	GD/none	N/A	N/A	63	0.01 (N:N/A)	N/A	1.55 (N:0.62–1.24)	N/A	N/A	2.4 (N: <1)	N/A	N/A	Chee [38]
F	22	China	N/A (mRNA)	No	1st	GD/none	N/A	N/A	5	0.01 (N:N/A)	>40 (N: 3.5–6)	5.43 (N:0.62–1.24)	N/A	N/A	5.8 (N: <1)	N/A	N/A	Chee [38]

Abbreviations: F: female; M: male; COVID-19: coronavirus disease 2019; AITD: autoimmune thyroid disease; GD: Graves’ disease; DM: diabetes mellitus; MMI: methimazole; N/A: not available; TSH: thyrotropin; fT3: free triiodothyronin. fT4: free tiroxine; TPOAb: anti-thyroid peroxidase antibody; TgAb: anti-thyroglobuline antibody; TRAb: TSH receptor antibody; US: ultrasound; Ref: reference.

**Table 3:** Overview of new cases and relapses of Graves’ disease following SARS-CoV-2 vaccination reported in the literature.

	New Onset GD	GD Recurrence
**Number of Cases**	48	15
**Sex**	F (70.8%)—M (29.2%)	F (73.3%)—M (26.7%)
**Age (years), median [IQR]**	43 [IQR 35–50.5]	41 [IQR 30–59]
**Type of SARS-Cov-2 vaccine**	BNT162b2 50% ChAdOx1 27% mRNA-1273 8.3% Ad26.COV2.S 2.2% Not specified (mRNA) 12.5%	BNT162b2 33.3% ChAdOx1 + CoronaVac 6.6% Ad26.COV2.S 6.6% CoronaVac 6.6% mRNA-1273 6.6% Not specified (mRNA) 40%
**Days to symptoms onset, median [IQR]**	10 [IQR 5–14]	11 [IQR 6–28]
**Major symptoms**	palpitations (61.9%) weight loss (35.7%) distal tremor (28.6%) behavioral/sleep disorders (26.1%) asthenia (21.4%) GI symptoms (14.3%)	palpitations (77.8%) sweating (55.5%) weight loss (33.3%)
**TSH (IU/mL), median [IQR]**	0.008 [0.004–0.01]	0.01 [0.008–0.01]
**fT3 (ng/L), median [IQR]**	13.2 [9.83–19.9]	13.5 [7.97–22.45]
**fT4 (ng/dL), median [IQR]**	3.58 [254–5.34]	3.32 [1.55–3.86]
**TRAb (IU/L), median [IQR]**	6.45 [4.39–16-56]	5.8 [3.85–13.4]

Abbreviations: GD, Graves’ disease; SARS-CoV-2, severe acute respiratory syndrome coronavirus 2; IQR, interquartile range; GI, gastrointestinal; TSH, thyrotropin; fT3, free triiodothyronine; fT4, free tiroxine; TRAb, TSH receptor antibody.

## Data Availability

The authors confirm that the data supporting the findings of this study are available within the article.
